# Traditional Chinese Medicine *ZHENG* Identification Provides a Novel Stratification Approach in Patients with Allergic Rhinitis

**DOI:** 10.1155/2012/480715

**Published:** 2012-04-18

**Authors:** Kai-Li Liang, Rong-San Jiang, Chia-Lin Lee, Pei-Jung Chiang, Jui-Shan Lin, Yi-Chang Su

**Affiliations:** ^1^Department of Otolaryngology, Taichung Veterans General Hospital, Taichung 40705, Taiwan; ^2^School of Medicine, Chung Shan Medical University, Taichung 40201, Taiwan; ^3^Department of Endocrinology and Metabolism, Taichung Veterans General Hospital, Taichung 40705, Taiwan; ^4^Department of Traditional Chinese Medicine, Taichung Veterans General Hospital, Taichung 40705, Taiwan; ^5^Graduate Institute of Chinese Medicine, College of Chinese Medicine, China Medical University, Taichung 40421, Taiwan

## Abstract

*Background*. We aimed to apply the *ZHENG *identification to provide an easy and useful tool to stratify the patients with allergic rhinitis (AR) through exploring the correlation between the quantified scores of AR symptoms and the TCM *ZHENGs*. *Methods*. A total of 114 AR patients were enrolled in this observational study. All participants received the examinations of anterior rhinoscopy and acoustic rhinometry. Their blood samples were collected for measurement of total serum immunoglobulin E (IgE), blood eosinophil count (Eos), and serum eosinophil cationic protein (ECP). They also received two questionnaire to assess the severity scores of AR symptoms and quantified TCM *ZHENG *scores. Multiple linear regression analysis was used to determine explanatory factors for the score of AR manifestations. *Results*. IgE and ECP level, duration of AR, the 2 derived TCM*ZHENG *scores of “*Yin*-*Xu − Yang*-*Xu*”, and “*Qi*-*Xu *+ *Blood*-*Xu*” were 5 explanatory variables to predict the severity scores of AR symptoms. The patients who had higher scores of “*Yin*-*Xu − Yang*-*Xu*” or “*Qi*-*Xu *+ *Blood*-*Xu*” tended to manifest as “sneezer and runner” or “blockers,” respectively. *Conclusions.* The TCM *ZHENG *scores correlated with the severity scores of AR symptoms and provided an easy and useful tool to stratify the AR patients.

## 1. Introduction

Allergic rhinitis (AR) is a common disease with a prevalence of at least 10% to 20% in western nations [1, 2]. Many patients suffering from AR seek help from complementary medical treatment, such as traditional Chinese medicine (TCM) [3–5]. Some well-designed controlled studies showed benefits of TCM treatment in allergic diseases [6–10]. Patients having the same disease in western medicine may have different TCM *ZHENG *diagnosis and therefore are prescribed different TCM herbal treatment.

*ZHENG* (syndrome), a basic unit and key concept in TCM theory, is the diagnosis made or concluded after careful analysis of all symptoms and signs. This process to achieve the diagnosis is based on the physiology and pathology of TCM, and is named “differentiation of *ZHENG*” or “*ZHENG* identification” [11]. This characteristic of TCM diagnosis denotes that the diseased person and the integrity of the human body are more focused than the person's disease. The purpose of collecting and analyzing symptoms and signs is to evaluate the overall maladjustment of the human body [12]. Guided with the *ZHENG* identification, the treatment modalities are individualized and mainly based on the* ZHENG* which each patient is diagnosed with.

Currently, there are no agreed ways to predict the severity of allergic rhinitis manifestations. Either nasal airway assessments, laboratory parameters, or physical findings usually show a low degree of correlation or a nonsignificant correlation with patients' symptoms [12–17]. Since the TCM* ZHENG* is diagnosed according to each patient's clinical manifestations, it reflects more subtle individual differences in patients with the same disease, which may be a tool to predict the severity of AR symptoms or categorize the AR patients. The purpose of this study was to probe the correlation between the severity of AR symptoms and the TCM *ZHENG*, and also try to adopt the TCM* ZHENG* identification to provide an easy and useful tool to stratify the AR patients.

## 2. Participants and Methods

### 2.1. Study Design and Subjects

This was an explorative, cross-sectional, and observational clinical trial. Patients diagnosed with AR were enrolled from the outpatient of the Otorhinolaryngology Department of Taichung Veterans General Hospital from 2004 to 2005. The diagnosis of AR was made by the specialist physician according to the clinical manifestations, history, and positive skin testing of a local screening panel (house dust, cotton, ragweed, *Candida, Alternaria, Aspergillus, Cladosporium*, and *Penicillium*). A written informed consent was obtained from each participant. Exclusion criteria for this trial were those who (1) took antihistamine, decongestant, or had used topical steroids within 2 weeks, (2) were under 12 years old, (3) were pregnant, (4) were ongoing immunotherapy, or (5) were with severe physical or mental illness. The study protocols were approved by the Institutional Review Board of the Taichung Veterans General Hospital (IRB TCVGH no. 930116/279).

### 2.2. Patient Assessments

#### 2.2.1. Physical Exam and Inflammatory Maker

One specialist physician (R. S. Jiang) performed the anterior rhinoscopy for all eligible patients and graded their rhinoscopic findings. The edematous degree of inferior turbinate and the amount of nasal discharge were graded from 0 (none) to 3 (severe). Nasal minimal cross-sectional area (MCA) was assessed by acoustic rhinometry in each patient. The blood samples were collected from the patients for analysis of the inflammatory marker, including: total serum immunoglobulin E (IgE), blood eosinophil count (Eos), and serum eosinophil cationic protein (ECP).

#### 2.2.2. Scoring of AR Symptoms Severity

Each enrolled patient completed a self-report questionnaire assessment to assess the severity of allergic symptoms within the latest 2 weeks: this questionnaire, which also included questions on age, gender, family history, comobility (allergic asthma, atopic dermatitis, or urticaria), and duration of AR, was specific to the severity of allergic symptoms including nasal obstruction, sneezing, rhinorrhea, itchy nose, and itchy eye. These symptoms were graded from 0 to 3 according to the severity within previous 2 weeks (0 = no symptom; 1 = mild symptom, no impact on daily life; 2 = moderate symptom, impact on daily life; 3 = severe symptom, impact on daily life).

#### 2.2.3. Scoring of TCM *ZHENGs*

In TCM, a disease is a common product of both pathogenetic factors and maladjustments in the body. The body must have the capacity to regulate itself in order to maintain homeostasis and adapt to the environmental stimulus. If the body's regulation ability fails to maintain homeostasis, then diseases may develop [12]. Therefore, the signs and symptoms expressed by patients are analyzed to identify the type of internal maladjustments (e.g., hyporesponse or hyperresponse). This diagnostic process is called “TCM *ZHENG* Identification.” The diagnosis of TCM *ZHENG* is the summary of a specific functional state of the human body [12]. There are many* ZHENGs* in TCM, either simple *ZHENG* or combined ones [11].

**Figure 1:**
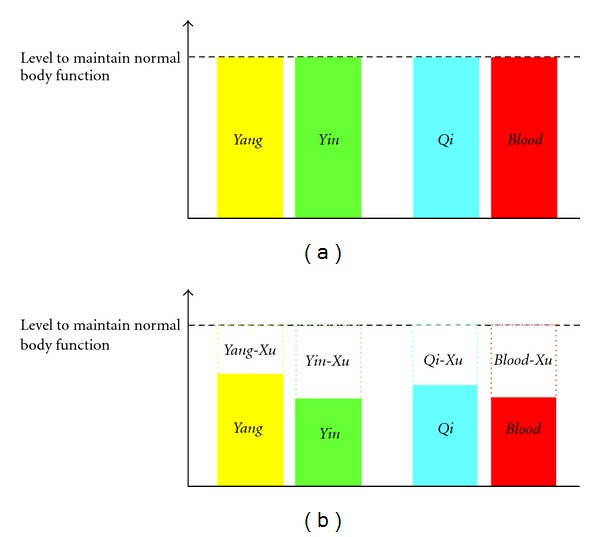
Illustration of the TCM *ZHENGs* and their derivative parameter. (a) Level of *Yang, Yin, Qi,* and *Blood* in normal physiological functional status. (b) Level of *Yang, Yin, Qi,* and *Blood* in pathological functional status.

In our study, the 4 basic TCM *ZHENGs*: “*Yin-Xu*, *Yang-Xu*, *Qi-Xu*, and *Blood-Xu*” were chosen to be measured in the AR patients. It was because *Internal Classis*, an important TCM literature, points out that: “*Qi, Blood, Yin,* and *Yang*” are 4 basic important elements to maintain the body's normal function; the physiological equilibrium and the circulation of *Qi* and *Blood* all change in response to the environmental variations. These responses help preserve the dynamic equilibrium of the body's *Yin* and *Yang *[18]. Once the functional status maintained by these 4 elements fails to keep its normal capacity, the body will manifest related signs and symptoms. Then, the 4 basic TCM *ZHENGs*: “*Yin-Xu*, *Yang-Xu*, *Qi-Xu*, and *Blood-Xu*” will be diagnosed (Figure 1).

In order to integrate TCM with modern medicine, each enrolled patient completed a self-report questionnaire assessment to score the 4 basic TCM *ZHENGs* (*Yin-Xu*, *Yang-Xu*, *Qi-Xu*, and *Blood-Xu*). The measurement of this questionnaire provided a quantified and comparable parameter to explore the correlation between TCM *ZHENGs* and the severity score of AR symptoms. An easy-applied and standardized TCM *ZHENG* diagnosis instrument was developed by our research team through 2 rounds of TCM experts' meetings. After several discussions, according to the TCM theory and considering its use in western medical setting, this instrument was designed to measure the 4 basic TCM *ZHENGs* by answering the 24 questions which described the signs and symptoms of the 4 *ZHENGs*. These signs and symptoms in the previous 2 weeks were self-reported and assessed by a 4-point frequency and intensity scale (each was graded from 0 to 3). With higher scores of frequency and intensity, a more pronounced pathological status of each TCM* ZHENG *was indicated (the TCM *ZHENG* Questionnaire and the TCM *ZHENG* measured by each question are listed in the appendix). The Cronbach *α* coefficients of this questionnaire were 0.70, 0.64, 0.77, and 0.76 for the four TCM *ZHENG* domains of “*Yin-Xu*, *Yang-Xu*, *Qi-Xu,* and *Blood-Xu*,*”* respectively. The scores of “*Yin-Xu*, *Yang-Xu*, *Qi-Xu,* and *Blood-Xu” *represented the decreased level of *Yin*, *Yang*, *Qi,* and *Blood* to maintain normal function of the body (Figure 1).

Furthermore, in TCM physiology, since “*Qi and Blood” *and* “Yin* and *Yang”* both work synergically to keep the equilibrium of the body, some items in the questionnaire were designed to measure more than one TCM *ZHENG*. Therefore, collinearity existed between the measurement results of “*Qi-Xu* and *Blood-Xu” and *“*Yin-Xu* and *Yang-Xu.”* To solve this problem, two derivative parameters were constructed based on not only the TCM theory about the pathological mechanism but also on the consideration of statistical modification: (1) “*Yin-Xu* − *Yang-Xu*”: this derived from the score of *Yang-Xu ZHENG *subtracted from the score of *Yin-Xu ZHENG*, and denoted the TCM *ZHENG “Yin-deficiency with Yang-hyperactivity.*” Physiologically, the normal body function is maintained upon the harmonious cooperation and mutual restrain of *Yin* and *Yang *[19]. Pathologically, once the decreased *Yin* is unable to restrain *Yang*, some signs and symptoms of *Yang-hyperactivity* will manifest, since the level of *Yin* and *Yang* is expected to be equal in normal circumstance. So, the derived parameter score of “*Yin-Xu* − *Yang-Xu*” was aimed to quantify this pathological status (Figure 1(b)).

(2) “*Qi-Xu* + *Blood-Xu”*: this derived from the score of *Qi-Xu ZHENG *added to the score of *Blood-Xu ZHENG*, and denoted the TCM *ZHENG “dual deficiency of Qi and Blood.*” In TCM physiology, *Blood* is transported by the driving force of *Qi* and they are both produced from the “middle energizer.” Pathologically, once the production of *Qi* and *Blood* or the efficacy of the transportation of *Qi* is decreased, a person may express signs and symptoms of “*Qi-Xu*” and/or “*Blood-Xu*.” So, the derived parameter score of “*Qi-Xu* + *Blood-Xu”* was aimed to quantify the whole body's pathological deficiency status of *Qi* and *Blood* (Figure 1(b)).

### 2.3. Statistical Analysis

Statistical analyses were conducted using the Statistical Package for the Social Science (SPSS Inc., Chicago, IL, USA) version 12.0. The demographic characteristics of the observed patients were described by frequency, percentage, mean, and standard deviation. The correlation among the study variables was examined by Spearmen's rank correlation. A test for linearity was used to evaluate the trend of the TCM *ZHENG* scores with the severity scores of AR symptoms. Multiple linear regression analysis with the total symptom score as the dependent variable was carried out to determine the variables independently associated with the severity of AR. Binary logistic regression was used to link the symptoms which impacted daily life (symptom score = 2 or 3) and TCM *ZHENG* score. Two-tailed *P* value <0.05 was considered statistically significant.

## 3. Results

A total of 114 AR patients were enrolled in our study. Both the questionnaire assessment and anterior rhinoscopy were done for all the 114 patients; examination of acoustic rhinometry for 111, measurements of IgE for 109, measurement of ECP for 106, and measurement of Eos for 100. The basic characteristics of the patients are listed in Table 1.

### 3.1. Correlation with the Severity Score of AR Symptoms

#### 3.1.1. Demographic Characteristics

There was no significant correlation between the AR patients' age and the total or each AR symptom score; neither the correlation between the smoking habit and the symptom score. Patients who had other allergic diseases (allergic asthma, urticaria, or atopic dermatitis) or family members (parents, grandparents, or siblings) with allergic diseases (allergic asthma, allergic rhinitis, urticaria, or atopic dermatitis) did not have higher symptom scores either. However, the duration of AR showed significant correlation with both the total symptom scores and the score of itchy eye (Table 2).

#### 3.1.2. Rhinoscopic Findings and Nasal Airway Assessment

There was no correlation noted between the scores of rhinoscopic findings and the AR symptom scores. The sum of second nasal minimum cross-sectional area (MCA2) measured by acoustic rhinometry did not correlate with the symptom scores either (Table 2).

#### 3.1.3. Inflammatory Makers

When correlation was analyzed between each two variables, IgE and Eos did not correlate to the total nasal symptom scores. When it goes to the individual nasal symptoms, the ECP level had significant correlation with the score of rhinorrhea (*r* = 0.206 and *P* = 0.034) and moderate correlation with sneezing (*r* = 0.164 and *P* = 0.093). The IgE level had moderate correlation with sneezing (*r* = 0.157 and *P* = 0.102) (Table 2).

#### 3.1.4. Scores of TCM *ZHENGs*

The score of “*Yin-Xu*” *ZHENG* correlated significantly with the total and each AR symptom score. The score of “*Yang-Xu*” *ZHENG* did not correlate with any symptom score. The scores of “*Qi-Xu*” and “*Blood-Xu*” *ZHENG* correlated significantly with the total symptom scores, the scores of nasal obstruction, and itchy nose and eye. 

The derivative parameter “*Yin-Xu* − *Yang-Xu*” correlated significantly with the total and each AR symptom score, except nasal obstruction. Another derivative parameter “*Qi-Xu* + *Blood-Xu”* correlated significantly with the total symptoms scores, the scores of nasal obstruction, and itchy nose and eye (Table 2).

### 3.2. Explanatory Factors for Severity Score of AR Symptoms

Then, multiple linear regression analysis was performed to determine explanatory (predictive) factors for the severity scores of AR symptoms. Beside the correlated variables noted in the above bivariate correlation analysis, since age and sex were important demographic factors, they were put into the multiple linear regression model. Simultaneously, IgE was also added considering it being checked regularly in the clinical practice for AR patients. 

In the beginning of the multiple linear regression analysis, we faced the problem of collinearity when the 4 basic TCM *ZHENG* scores were used as explanatory variables for regression analysis. The collinearity was solved by using the score of the derivative parameter “*Yin-Xu* − *Yang-Xu*” and “*Qi-Xu* + *Blood-Xu”* instead of the 4 basic TCM *ZHENGs* for the regression model.

Finally, we found 5 independent predictors: IgE level (*P* = 0.039), ECP level (*P* = 0.017), duration of AR (*P* = 0.016), the scores of “*Yin-Xu* − *Yang-Xu*” (*P* = 0.004), and the score of “*Qi-Xu* + *Blood-Xu”* (*P* = 0.015) (using enter regression model, *R* ^2^ = 0.280, *P* < 0.001, Table 3).

### 3.3. Correlation between Predictive Factors and Each Symptom

Furthermore, we went on to exam the correlation between the above 5 predictors and each AR symptom using binary logistic regression. Before the analysis, the AR symptom scores were processed in advance as follows: (1) the original symptom scores graded from 0 to 1 were recategorized into “0,” which meant no impact on daily life; (2) the original scores graded from 2 to 3 were recategorized into “1,” which meant the symptoms had impact on daily life.

When binary logistic regression was used to link these predictors with the AR symptoms which impacted daily life, we found that nasal obstruction which impacted daily life was correlated with higher scores of “*Qi-Xu* + *Blood-Xu”* (OR = 1.081, 95% CI = 1.009 to 1.158). The symptoms of rhinorrhea and itchy nose which impacted daily life were correlated with higher scores of “*Yin-Xu* − *Yang-Xu*” (OR = 1.165, 95% CI = 1.018 to 1.334, and OR = 1.147, 95% CI = 1.012 to 1.300, resp.). The symptom of itchy eye which impacted daily life (the score of itchy eye = 2 or 3) was correlated with higher scores of “*Yin-Xu* − *Yang-Xu*” (OR = 1.164, 95% CI = 1.015 to 1.335) and longer duration of AR (OR = 1.015, 95% CI = 1.006 to 1.024) (Table 4).

## 4. Discussion

This explorative, cross-sectional, and observational clinical study adopted and integrated both the diagnostic method of western medicine and TCM in patients with AR. AR is defined as a symptomatic disorder of the nose induced after allergen exposure by an IgE-mediated inflammation. The update treatment guidelines initiated by the World Health Organization recommend classification of allergic rhinitis into “intermittent” (IAR) or “persistent” (PER) allergic rhinitis, instead of previous classification of “seasonal” or “perennial” allergic rhinitis [1, 2]. It is believed that the new classification shows better adherence to real life. In this study, we enrolled patients with history of typical symptoms of allergic rhinitis including nasal obstruction, sneezing, rhinorrhea, itchy nose and eyes. The IgE-mediated etiology of the enrolled rhinitis patients has confirmed with positive skin testing of a local screening panel. Therefore we enrolled a group of rhinitis patients with same underlying etiology (IgE-mediated allergic rhinitis). We excluded the patients who took antihistamine, decongestant or had used topical steroids within 2 weeks, and who were ongoing immunotherapy. The above exclusion criteria were for reducing the drug effects affecting our assessment.

TCM doctors diagnose the *ZHENG* based on the TCM theory after inquiry and physical examination. However, the theory of TCM is complicated and not easily realized by western medicine (WM) physicians and investigators. WM doctors are often skeptical about the validity of TCM clinical diagnosis [20, 21]. We designed the “TCM *ZHENG* Questionnaire” as a simplified mathematic model of TCM inquiry and provided an easy-applied, standardized diagnosis tool. Questions in the questionnaire were designed to survey patients' physical conditions based on the four basic TCM *ZHENGs: “Yin-Xu*, *Yang-Xu*, *Qi-Xu*, and *Blood-Xu*.*”* Higher scores in the questionnaire meant more pronounced pathological statuses. The TCM physical findings of patients' pulse, tongue, nails, lips, and face were not included in this study because of the difficulty in standardization.

Investigations of the predictors of the severity of allergic rhinitis manifestations are few and conflicting. Several studies have proved the relationship between IgE, ECP and Eos, and atopic diseases [22–25]. However, using inflammatory markers as predictors for AR's severity has not been established [13, 14, 26, 27]. Winther et al. [14] conducted a study and investigated the relationship between laboratory parameters and the severity of AR. They found that certain laboratory parameters were significantly correlated with disease severity, but could account for only a minor part of the seasonal variation of the symptom scores. In this study, we found only the duration of AR and the TCM *ZHENG* scores to be associated with the severity of AR by analysis of bivariate correlation. The inflammatory parameters correlated with the total symptom scores after adjusting the TCM *ZHENG* scores, meaning the TCM *ZHENG* scores were a confounding factor to the inflammatory parameters. 

Acoustic rhinometry is a geographic measurement of the nasal cavity by using reflections of sound wave. The acoustic rhinometry is safe and its validity has been proven by comparison with measures obtained by computerized tomography or magnetic resonance imaging scanning [28–31]. However, the subjective reporting of nasal obstruction may not correlate well with acoustic rhinometry measures [17, 32]. This could be because the sensation of nasal obstruction can be influenced by changes in the ostiomeatal complex and existence of nasal discharge rather than purely reflecting nasal cavity size [15]. We found the result of acoustic rhinometry had no significant correlation with the total symptom scores or the scores of nasal obstruction.

The TCM *ZHENG* (“*Yin-Xu* − *Yang-Xu*” and “*Qi-Xu* + *Blood-Xu”*), duration of AR, IgE, and ECP level were found to be good predictors for the severity scores of AR in our study. However, the *R* ^2^ value was only 0.28, indicating some other factors could contribute to the severity of allergic rhinitis. We believe that environmental factors, or mucociliary function may play a role causing the severity of AR manifestations. 

Khanna and Shah [33] reported a new classification of patients with allergic rhinitis, according to the ARIA report [1], as “sneezer and runner” and “blocker” was mandatory. Their study demonstrated that the two groups had distinct clinical profiles. We also found that these two groups had different TCM *ZHENG* scores: “blockers” (the symptom scores of nasal obstruction = 2 or 3) having significant higher scores of “*Qi-Xu* + *Blood-Xu”*, while “sneezer and runner” (the symptom scores of rhinorrhea, itchy nose or eye = 2 or 3) having higher scores of “*Yin-Xu* − *Yang-Xu*”. From the TCM pathological point of views, these findings were very reasonable and closely fitted to the TCM theory. Since the nasal obstruction may be caused by the deficiency of *Qi* and/or *Blood*; while the rhinorrhea or itchy nose or eye are the manifestations of *Yin-deficiency with Yang-hyperactivity*. These results showed that the TCM *ZHENG* diagnosis correlated with the modern western medicine, and the TCM *ZHENG* diagnosis could reflect subtle differences among the patients with AR. 

To our knowledge, this was the first study which adopted the TCM diagnostic questionnaires to quantify disease-specific severity and to categorize the patients with AR. Our results revealed that the TCM diagnostic questionnaires can be used similarly to disease specific quality of life standardized questionnaires such as the SF-36 (a general QOL questionnaire) or the RQLQ (a disease-specific QOL) instrument. Our research team had conducted several clinical studies which adopted both the TCM diagnostic questionnaires and the quality of life standardized questionnaires. It was found that the results measured by the 2 questionnaires were comparable in several aspects. Our team will report these interesting findings and new application of TCM diagnosis continuously.

## 5. Conclusion

The TCM *ZHENG *score, the duration of AR, and the IgE and ECP level were found to be independently and significantly explanatory of the severity of AR manifestations. The TCM *ZHENG* diagnosis correlated with the modern western diagnosis and may provide a novel approach to stratify the AR patients. These findings may provide a new applied field of TCM* ZHENG* diagnosis.

## Figures and Tables

**Table 1:** Basic characteristic of the observed patients (*n* = 114).

	Number (percentage)	Mean ± SE
Age (yr)		27.80 ± 1.19
Gender	
Male	63 (55.3%)
Female	51 (44.7%)	
Smoking	17 (14.9%)	
Family history ^a^	79 (69.3%)	
Comobility^b^	28 (24.6%)	
Duration of AR (months)		93.75 ± 6.86

Rhinoscopic findings	Turbinate swelling	1.86 ± 0.06
	Nasal discharge	0.71 ± 0.07
	Total scores	2.57 ± 0.09
Sum of MCA2 (cm^2^)		0.87 ± 0.03

Inflammatory markers	IgE (kU/L)	407.60 ± 66.01
	ECP (pg/mL)	18.89 ± 2.01
	Eos (/mm^3^)	279.35 ± 19.65

Symptom scores	Nasal obstruction	2.04 ± 0.08
	Sneezing	1.86 ± 0.08
	Rhinorrhea	2.11 ± 0.08
	Itchy nose	1.49 ± 0.08
	Itchy eye	1.30 ± 0.09
	Total symptom scores	8.81 ± 0.29

Scores of *ZHENG *	*Yin-Xu *	6.25 ± 0.33
	*Yang-Xu*	5.39 ± 0.30
	*Qi-Xu*	7.25 ± 0.38
	*Blood-Xu*	7.96 ± 0.40
	*Yin-Xu * ** − ** *Yang-Xu* ^ c^	0.86 ± 0.35
	*Qi-Xu* + *Blood-Xu * ^d^	15.21 ± 0.73

^a^Parents, grandparents, or siblings had allergic rhinitis, allergic asthma, atopic dermatitis, or urticaria.

^b^Patients had allergic asthma, atopic dermatitis, or urticaria.

^c^Score of *Yang-Xu ZHENG *subtracted from score *Yin-Xu* of *ZHENG.*

^d^Score of *Qi-Xu ZHENG *added to score of *Blood-Xu ZHENG.*

AR: allergic rhinitis; MCA2 = the second minimal cross-sectional area.

**Table 2:** Correlations of study variables.

Symptom score	Total	Nasal obstruction	Sneezing	Rhinorrhea	Itchy nose	Itchy eyes
Age	0.064	0.103	0.067	0.011	0.047	0.011
Smoking	−0.115	−0.095	−0.050	−0.128	−0.040	−0.085
Comobility^a^	0.104	0.063	0.132	0.119	0.060	0.026
Duration of AR	0.243*	0.109	0.133	0.123	0.172	0.312*
Rhinoscopic findings	0.117	0.110	0.014	0.108	−0.110	0.107
MCA2	−0.109	−0.063	−0.085	−0.039	−0.156	−0.070
IgE	0.081	−0.180	0.157	0.064	0.029	0.049
ECP	0.116	−0.022	0.164	0.206*	0.119	−0.018
Eos	0.120	−0.600	0.126	0.156	0.101	0.037
*Yin-Xu*	0.357*	0.206*	0.188*	0.200*	0.352*	0.340*
*Yang-Xu*	0.730	0.128	−0.810	−0.180	0.0780	0.131
*Qi-Xu*	0.256*	0.199*	0.054	0.012	0.342*	0.304*
*Blood-Xu*	0.267*	0.194*	0.097	0.074	0.281*	0.332*
*Yin-Xu * ** − ** *Yang-Xu*	0.282*	0.082	0.259*	0.238*	0.245*	0.196*
*Qi-Xu* + *Blood-Xu *	0.266*	0.194*	0.065	0.036	0.325*	0.339*

Data presented with coefficient.

*P  value < 0.05.

^a^Patients had allergic asthma, atopic dermatitis, or urticaria.

**Table 3:** Factors predicting severity scores of symptom in patients with allergic rhinitis.

Variable	Regression coefficient	SE	*t*
Age	0.017	0.024	0.701
Male sex	−0.995	0.589	−1.689
Duration of AR	0.01*	0.004	2.451
IgE	0.0008*	0.000	2.091
ECP	0.033*	0.014	2.418
*Yin-Xu * ** − ** *Yang-Xu*	0.232*	0.078	2.957
*Qi-Xu* + *Blood-Xu *	0.092*	0.037	2.480

Model: multiple linear regression, use enter regression; *R* ^2^ = 0.280, *P* < 0.001.

*P < 0.05.

**Table 4:** Link of allergic symptoms which impacted daily life (symptom score = 2 or 3) with predictive factors.

	Nasal obstruction		Sneezing		Rhinorrhea		Itchy nose		Itchy eyes	
	Model of regression: enter		Model of regression: enter		Model of regression: enter		Model of regression: enter		Model of regression: enter	
	*R* ^2^ = 0.122, *P* = 0.244		*R* ^2^ = 0.150, *P* = 0.101		*R* ^2^ = 0.210, *P* = 0.022		*R* ^2^ = 0.161, *P* = 0.063		*R* ^2^ = 0.323, *P* < 0.001	
	Accuracy of model = 74.0%		Accuracy of model = 67.3%		Accuracy of model = 68.3%		Accuracy of model = 61.5%		Accuracy of model = 71.2%	
	OR	95% CI	OR	95% CI	OR	95% CI	OR	95% CI	OR	95% CI
Age	1.009	0.971–1.049	1.014	0.976–1.054	1.011	0.970–1.053	1.012	0.976–1.049	0.994	0.953–1.036
Male sex	1.425	0.521–3.894	1.313	0.514–3.353	1.393	0.503–3.860	1.470	0.604–3.579	2.096	0.796–5.522
Duration of AR	1.002	0.995–1.008	1.003	0.996–1.010	1.007	0.999–1.015	1.02	0.995–1.008	1.015*	1.006–1.024
IgE	1.001	0.999–1.002	1.001	1.000–1.003	1.001	1.000–1.002	1.000	1.000–1.001	1.000	1.000–1.001
ECP	1.015	0.987–1.043	1.021	0.992–1.051	1.026	0.992–1.061	1.009	0.988–1.030	1.017	0.994–1.040
*Yin-Xu * ** − ** *Yang-Xu*	1.003	0.878–1.147	1.111	0.982–1.256	1.165*	1.018–1.334	1.147*	1.012–1.300	1.164*	1.015–1.335
*Qi-Xu* + *Blood-Xu *	1.081*	1.009–1.158	1.020	0.961–1.083	1.028	0.961–1.098	1.056	0.996–1.119	1.055	0.992–1.123

Analyzed by binary logistic regression.

OR: odds ratio.

95% CI: 95% confidence interval.

*P < 0.05.

**Table 5:** 

	0	1	2	3	TCM* ZHENG measured *
(1) I have become fretful and irritated about everything.	□	□	□	□	*Yin-Xu*
(2) I have heartburn.	□	□	□	□	*Yin-Xu*
(3) I have been suffering from insomnia.	□	□	□	□	*Yin-Xu Blood-Xu*
(4) It takes a long time for me to fall asleep.	□	□	□	□	*Yin-Xu Blood-Xu*
(5) My hands and feet are warm.	□	□	□	□	*Yin-Xu*
(6) I have night sweat even if it's not hot.	□	□	□	□	*Yin-Xu*
(7) I'm always thirsty. My throat feels completely dried out soon after I drink.	□	□	□	□	*Yin-Xu*
(8) I have to get up to pee at night.	□	□	□	□	*Yang-Xu *
(9) I urinate a lot, and the color of my urine is faint.	□	□	□	□	*Yang-Xu *
(10) I feel cold when others feel cool and comfortable.	□	□	□	□	*Yang-Xu *
(11) I wear more layers because I feel cold.	□	□	□	□	*Yang-Xu *
(12) I like to stay in a warm place and like to huddle up to feel warm.	□	□	□	□	*Yang-Xu *
(13) I have loose stools.	□	□	□	□	*Yang-Xu *
(14) I feel very tired after mild activity.	□	□	□	□	*Qi-Xu *
(15) I feel dizzy when getting up quickly.	□	□	□	□	*Qi-Xu Blood-Xu *
(16) I do not like to talk because I soon feel tired after saying a few words.	□	□	□	□	*Qi-Xu *
(17) I still feel sleepy after a long sleep.	□	□	□	□	*Qi-Xu *
(18) I feel lightheaded.	□	□	□	□	*Qi-Xu Blood-Xu *
(19) I sweat even if it is cool.	□	□	□	□	*Qi-Xu *
(20) I get out of breath when I walk a little.	□	□	□	□	*Qi-Xu *
(21) I feel palpations even when still or peaceful.	□	□	□	□	*Blood-Xu *
(22) I am neither nearsighted nor farsighted (or has already been corrected), but I still have blurred vision.	□	□	□	□	*Blood-Xu *
(23) My body and limbs feel numb when I keep still.	□	□	□	□	*Blood-Xu *
(24) My ears ring when it is quiet.	□	□	□	□	*Blood-Xu*

## Appendix

### A. Traditional Chinese Medicine *ZHENG* Questionnaire

In Table 5 you will find a list of symptoms associated with the traditional Chinese medicine *ZHENG*. We would appreciate you answering the following questions to the best of your ability. Please rate your problems as they have been over *the past two weeks* (all questions are graded as 0 = never; 1 = sometimes; 2 = often; 3 = always).
